# Plasmablastic Lymphoma Presenting With Rectosigmoid Perforation in a Human Immunodeficiency Virus-Positive Patient

**DOI:** 10.7759/cureus.24964

**Published:** 2022-05-13

**Authors:** Muhammad H Zafar, Lola C Gil, Saman Karimi, Saad Arain, Bindu Niravel, Jessica Martinolich, John Galvin, Carlos A Murga-Zamolloa, Gerald Gantt Jr.

**Affiliations:** 1 Department of Colorectal Surgery, University of Illinois at Chicago, Chicago, USA; 2 Department of Pathology, University of Illinois at Chicago, Chicago, USA; 3 Department of Hematology and Oncology, University of Illinois at Chicago, Chicago, USA; 4 Department of Colorectal Surgery, Cook County Health and Hospital System, Chicago, USA

**Keywords:** stem cell transplant, open anterior resection, perforation, hiv, plasmablastic lymphoma

## Abstract

Plasmablastic lymphoma (PBL) is a rare variant of diffuse large B-cell lymphoma (DLBCL) associated with human immunodeficiency virus (HIV)-positive patients. It accounts for only 2% of all acquired immune deficiency syndrome (AIDS)-related lymphomas (ARLs). We present the case of a 45-year-old male who presented to the emergency department (ED) with a three-month history of abdominal pain, diarrhea, and unintentional 50-lb weight loss. On an earlier presentation to the ED three months prior, the patient was diagnosed with norovirus and *Helicobacter pylori* infection and received outpatient treatment without resolution of his symptoms. This prompted further investigation with a CT of the abdomen and pelvis with IV contrast that revealed severe sigmoid colitis with pneumoperitoneum and a pericolonic air-containing fluid collection, consistent with a contained perforation with abscess formation. He was admitted, resuscitated, and initially treated with antibiotics and parenteral nutrition. The patient underwent a laparoscopic converted to open anterior resection with end colostomy. Pathology revealed HIV-related PBL. He was subsequently treated with dose-adjusted etoposide, prednisone, vincristine, cyclophosphamide, doxorubicin, and rituximab (DA-EPOCH-R) chemotherapy regimen and an autologous stem cell transplant. Despite its rare association with HIV, PBL should be considered a differential diagnosis for HIV-positive patients who present with gastrointestinal (GI) pathology, and additional investigations should be conducted if symptoms do not resolve despite appropriate medical management at the time.

## Introduction

The gastrointestinal (GI) tract is the most common extranodal site for lymphoma, accounting for about 5%-40% of all cases. Of these cases, only 1%-4% are primary gastrointestinal lymphomas, making these entities extremely rare [1].

Plasmablastic lymphoma (PBL) is an aggressive and rare variant of diffuse large B-cell lymphoma (DLBCL), which has a greater prevalence in immunosuppressed patients and those who are living with human immunodeficiency virus (HIV) and Epstein-Barr virus (EBV) infections [2]. While PBL is considered an acquired immune deficiency syndrome (AIDS)-defining illness, it only accounts for approximately 2% of all AIDS-related lymphomas (ARLs) [3].

Despite these associations, the literature does describe rare cases of PBL occurring in patients who harbor none of these infections. Also, there are certain differences that have been reported in the clinical and pathological characterization of PBL between HIV-positive and HIV-negative patients. PBL patients living with HIV tend to be younger, predominantly male, and have more frequent oral involvement. They also have a significantly higher expression of CD20, CD56, and EBV-encoded RNA compared with HIV-negative patients [4].

The prognosis of PBL is generally poor. A systematic review found that HIV-positive patients have a median survival rate of 15 months compared to nine months for patients with an HIV-negative status [5]. Due to the paucity of PBL cases, not only is it difficult to evaluate its true prevalence and incidence, but establishing a chemotherapy regimen that would be the standard of care for PBL has also proved to be challenging. We present the case of a male who presented with a perforated rectosigmoid PBL.

We present the following article/case in accordance with the CARE guidelines (for CAse REports), which were developed by an international group of experts with the aim of facilitating an increase in the accuracy, transparency, and usefulness of case reports.

## Case presentation

The patient is a 45-year-old male with a significant past medical history of HIV, diagnosed in 2012, who had deferred highly active antiretroviral therapy (HAART) treatment until two months prior to his acute presentation. The patient was being treated with elvitegravir/cobicistat/emtricitabine/tenofovir alafenamide at the time of presentation. He presented to the emergency department with a three-month history of abdominal pain, non-bloody diarrhea, and unintentional 50-lb weight loss. He had initially presented as an outpatient three months prior and was diagnosed and treated for norovirus and *Helicobacter pylori* infections. The patient had no resolution of his symptoms during this three-month period. Delay in further evaluation coincided with the beginning of the coronavirus disease 2019 (COVID-19) pandemic. He had no previous colonoscopy and no family history of colorectal malignancy or inflammatory bowel disease.

Clinical findings

He was afebrile on presentation with normal vital signs. Positive findings on examination included tenderness on palpation in the left lower quadrant and lower-mid abdomen without signs of peritonitis.

Timeline

After initial evaluation in the emergency department, the patient was admitted and started on piperacillin/tazobactam and parenteral nutrition given his profound weight loss. On day 2 of his admission, a CT scan of the abdomen/pelvis revealed a contained colonic perforation that prompted the Department of Colorectal Surgery to be consulted. Given the CT scan findings and persistence of the patient’s abdominal pain and diarrhea, the team decided to intervene surgically. During his inpatient admission, the patient did not display any signs of peritonitis, hemodynamic instability, or leukocytosis. Therefore, on day 6 of his admission, the patient underwent an elective laparoscopic converted to open Hartmann’s procedure. Following surgery, the patient continued to recover well and was discharged on postoperative day 6.

Diagnostic assessment

Laboratory values on arrival were significant for a CD4 count of 266 cells/µL, hemoglobin of 9.6 g/dL, 29.2% hematocrit, and white blood cell count of 7.6 × 10^3^/µL with a normal differential (Table 1).

**Table 1:** Laboratory characteristics at initial presentation to the emergency department for rectosigmoid perforation.

Laboratory characteristics	Results	Reference values
Hemoglobin (g/dL)	9.6	13.2-18
Hematocrit (%)	29.2	38-55
White blood cell count (thousands/µL)	7.6	3.9-12
CD4 count (cell/µL)	266	438-1,501

**Figure 1:**
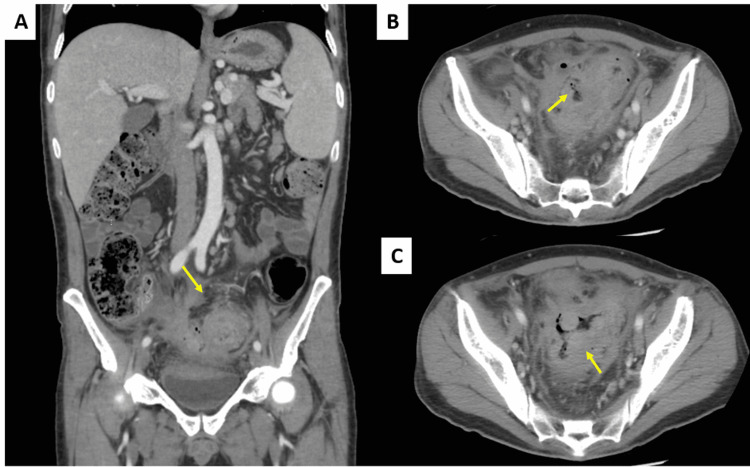
(A) Severe wall thickening and pericolonic inflammatory changes in the sigmoid colon (arrow). (B) Pericolonic extraluminal air and fluid (arrow). (C) Air-containing fluid collection, consistent with contained perforation with abscess formation (arrow).

**Figure 2:**
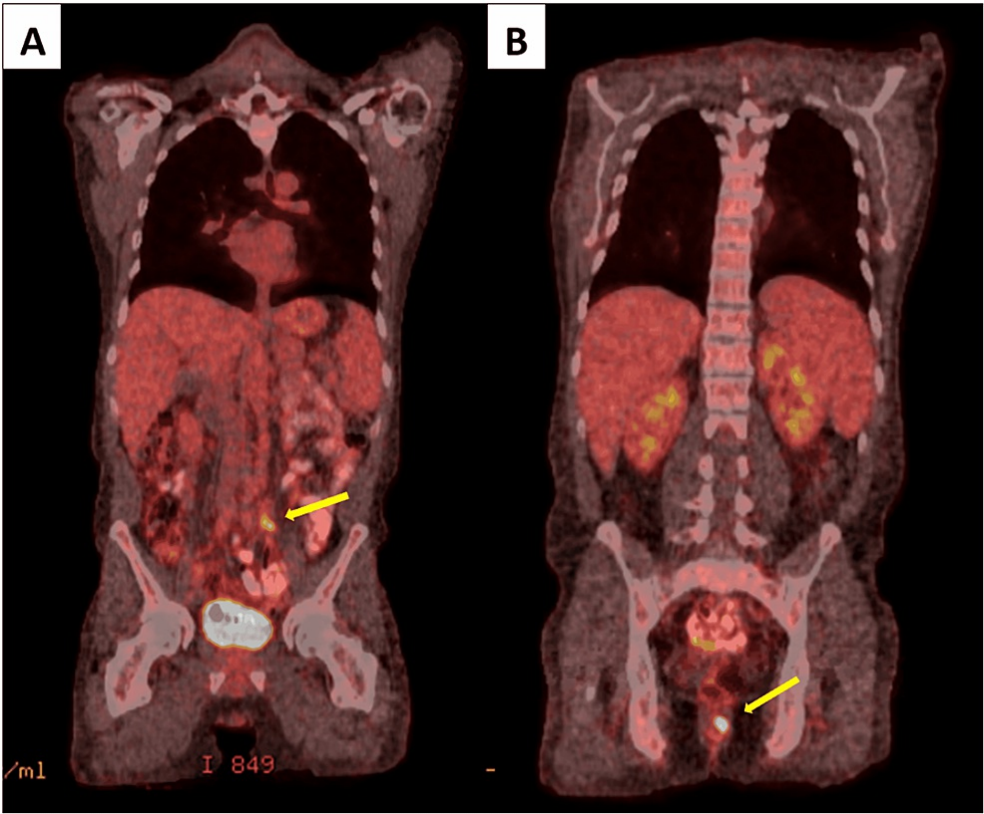
(A) Focus of high FDG avidity adjacent to the left common iliac artery, suspicious for metastatic disease (arrow). (B) Focus of high FDG avidity within the rectum, suspicious for malignancy (arrow).

A CT scan of the abdomen/pelvis with IV contrast revealed sigmoid colitis with pericolonic pneumoperitoneum and an adjacent air-containing fluid collection, consistent with contained perforation with abscess formation (Figure 1). An underlying mass could not be excluded. A PET/CT performed in a subsequent visit was positive for high FDG activity in both the rectum and adjacent to the left common iliac artery, suspicious for malignancy and metastatic disease (Figure 2).

On day 6 of his admission, the patient underwent a laparoscopic converted to open Hartmann’s procedure, cystoscopy with bilateral ureteral stent placement. The cystoscopy revealed inflammation of the posterior wall of the bladder without evidence of fistula. Operative findings included a significant inflammatory process involving the distal sigmoid and proximal rectum with dense adhesions involving the terminal ileum and pelvic sidewall. Opening the specimen revealed a large polypoid lesion at the distal end of a thickened obstructed bowel with evidence of diverticula.

**Figure 3:**
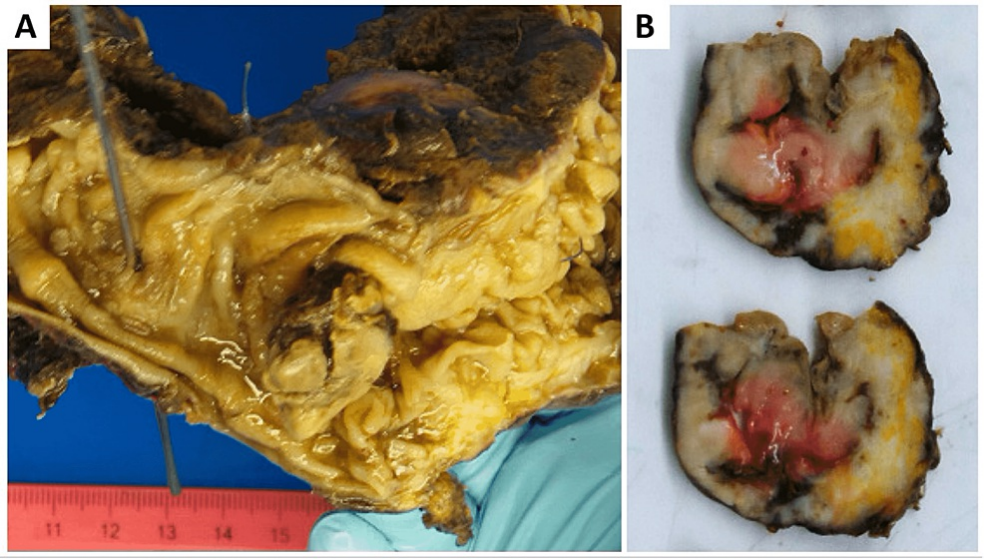
Sigmoid and proximal rectum segmentectomy. (A) A tan-brown thickened colonic mucosa with a transmural perforation site. (B) Cross section of the sigmoid colon revealing a soft, pink, hemorrhagic area below the thickened mucosa with diffuse fibrosis of the colonic wall.

Gross pathology evaluation of the surgical specimen around the area of perforation was remarkable for thickening of the mucosa, with no definitive ulcerations or polypoid lesions (Figure 3). Histologic examination revealed diffuse lymphoid infiltrates with panmural extension; the lymphoid infiltrates were predominantly composed of medium to large atypical lymphocytes with plasmablastic features, with open chromatin and prominent nucleoli. Adjacent areas of necrosis were also present. Sixteen mesenteric lymph nodes were harvested; all were negative for malignancy. The proximal and distal resection margins were negative.

**Figure 4:**
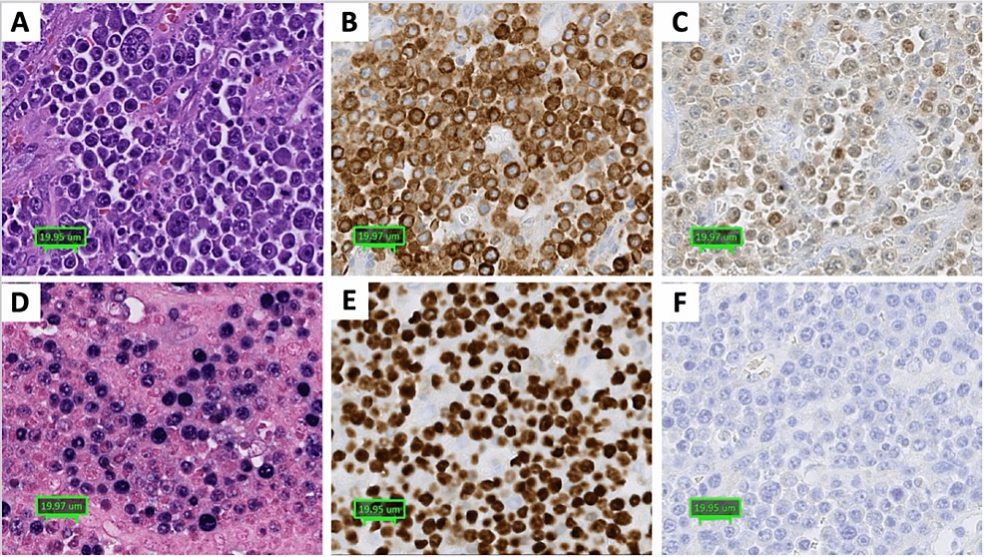
Immunohistochemical phenotype. (A) High magnification of hematoxylin and eosin (H&E) staining of the atypical lymphoid infiltrates demonstrating immunopositivity for (B) CD79a and (C) MUM-1. (D) In situ hybridization demonstrating EBER RNA expression. The atypical lymphoid infiltrates also demonstrate (E) >90% proliferation index by Ki67 immunostaining and (F) lack of immunoreactivity to CD138.

Immunohistochemical stains were performed and demonstrated that the atypical lymphoid infiltrates were positive for CD45, MUM-1, CD79a, and BCL-2, with weak CD20 positivity in approximately 10% of the neoplastic cells. In situ hybridization (EBER) was positive in the tumor cells for EBV. The lymphoid infiltrates were negative for PAX-5, cyclin D-1, CD10, BCL-6, CD3, CD5, CD138, CAM5.2, SOX-10, and MCK. The Ki-67 proliferation index was more than 90% within the atypical lymphocytes (Figure 4).

Although CD138 negativity is rare in plasmablastic lymphomas, the overall morphological findings, immunohistochemical profile characterized by negative PAX-5, weak/negative CD20, positive MUM-1, high Ki-67 proliferation index, EBV-positive status, and clinical context support the classification as plasmablastic lymphoma [6].

There was adequate access to testing, and no other financial or cultural diagnostic challenges were faced during all stages of the patient’s diagnostic workup.

Therapeutic intervention

The patient had a close follow-up with the Department of Hematology and Oncology to discuss further workup, which included a bone marrow biopsy, PET/CT, and lumbar puncture. His bone marrow biopsy was noted to be negative for involvement by a lymphoproliferative neoplasm. His PET/CT was notable for a focus of high FDG avidity within the rectum and an area adjacent to the left common iliac artery suspicious for malignant involvement of a lymph node (Figure 2). Besides mild FDG avidity within the bilateral inguinal lymph nodes that were felt to be possibly infectious/inflammatory, there were no other areas concerning for malignancy. A diagnostic lumbar puncture was performed and was noted to be negative. Thus, he was staged as a IIE PBL per the Lugano classification.

The patient was subsequently started on treatment with dose-adjusted etoposide, prednisone, vincristine, cyclophosphamide, doxorubicin, and rituximab (DA-EPOCH-R) and consolidation with BCNU, etoposide, cytarabine, and melphalan (BEAM) and autologous stem cell rescue.

Follow-up and outcomes

The patient was followed up two months after completing stem cell transplantation. He is active with occasional fatigue. During the course of his treatment, the patient has been compliant with both follow-up and the recommended medication regimen, tolerating the medications well.

## Discussion

Combination antiretroviral therapy (cART) has improved the outcomes of HIV-infected individuals in a myriad of ways, one of which has been to decrease the incidence of AIDS-related lymphomas (ARLs). Between 1996 and 2000, the incidence of ARLs was 449 cases per 100,000 person-years, and this has decreased to 194 cases per 100,000 person-years in the time period between 2006 and 2010 [7]. Despite this decline in ARL incidence, it is still important for clinicians to have a high index of suspicion for lymphoma in a patient with HIV/AIDS.

Primary GI lymphomas are so scarce in practice that no single institution can possibly gather enough data to put forth guidelines for treatment, diagnosis, or prognosis criteria for these cancers. The Dawson criteria were created after evaluation of 37 cases over the course of 25 years to clinically help clinicians diagnose primary intestinal malignancies after a history of false labeling many malignant lymphoid tissue tumors as such. The Dawson criteria consider a primary intestinal malignancy under the following circumstances: (1) the absence of peripheral lymphadenopathy, (2) lack of enlarged mediastinal lymph nodes, (3) normal total and differential WBC, (4) predominance of bowel lesion at the time of laparotomy, and (5) no lymphomatous involvement of the liver/spleen. Following the Dawson criteria, we can establish the diagnosis of our patient as a primary GI malignancy even after its unusual presentation [8]. In particular, PBL can have a presentation that is different from typical lymphomas; specifically, it is characterized by more extranodal gastrointestinal involvement and uncommonly presents with lymphadenopathy. B symptoms (fevers, weight loss, and drenching night sweats) are still typical [9]. A lack of recognition of this atypical presentation can lead to delayed diagnosis and treatment, so a high index of suspicion for HIV-infected individuals with B symptoms and GI symptoms is required. This especially includes patients who may be afflicted by an infectious GI pathogen but still have persistent symptoms despite treatment. With greater clinical awareness, we may potentially detect a higher prevalence of this disease, which may improve research and therefore treatment options for this type of lymphoma.

The high mortality rates and paucity of research on adequate treatment make the high clinical suspicion for and early recognition of PBL even more important. Because of its rarity, high-quality randomized clinical trials investigating the optimum treatment are lacking. However, due to its aggressive nature, the standard cyclophosphamide, doxorubicin, vincristine, and prednisone (CHOP) therapy alone is considered inadequate. Thus, more intensive regimens such as DA-EPOCH, hyper-fractionated cyclophosphamide, vincristine, doxorubicin, dexamethasone, and high-dose methotrexate and cytarabine (Hyper-CVAD-MA), and cyclophosphamide, vincristine, doxorubicin, high-dose methotrexate/ifosfamide, etoposide, and high-dose cytarabine (CODOX-M/IVAC) are recommended [8-10].

Although CD20 positivity is uncommon in PBL, it was noted to be weakly expressed in our patient’s malignancy. Thus, rituximab, an anti-CD20 antibody, was added to his regimen. Additionally, due to its aggressive nature, high-dose chemotherapy plus autologous stem cell rescue is often recommended in the first complete remission. Because our patient is otherwise young and fit, he was treated with an intensive chemotherapy regimen and autologous stem cell transplant.

## Conclusions

In conclusion, PBL should be considered a differential diagnosis for patients with HIV with gastrointestinal pathology, and additional investigations should be conducted if symptoms do not resolve as expected. Moreover, colorectal lymphomas can present as fungating, ulcerative, infiltrative, or combinations of these and can be located anywhere in the gastrointestinal tract from the mouth to the anus. Lastly, high-risk patients should be encouraged to seek medical care despite concerns of the COVID-19 pandemic, as an increase in morbidity and mortality due to delayed care may outweigh the risk of seeking medical attention.
